# *Mycobacterium ulcerans* and Other Mycolactone-Producing Mycobacteria Should Be Considered a Single Species

**DOI:** 10.1371/journal.pntd.0000663

**Published:** 2010-07-27

**Authors:** Sacha J. Pidot, Kingsley Asiedu, Michael Käser, Janet A. M. Fyfe, Timothy P. Stinear

**Affiliations:** 1 Department of Microbiology and Immunology, University of Melbourne, Melbourne, Australia; 2 Department of Microbiology, Monash University, Clayton, Australia; 3 World Health Organization, Geneva, Switzerland; 4 Swiss Tropical and Public Health Institute, Basel, Switzerland; 5 University of Basel, Basel, Switzerland; 6 Victorian Infectious Diseases Reference Laboratory, North Melbourne, Australia; Kwame Nkrumah University of Science and Technology (KNUST) School of Medical Sciences, Ghana

The nomenclature of *Mycobacterium ulcerans* has become confused with the discovery that other mycobacteria that are not necessarily associated with Buruli ulcer also produce the lipid toxin mycolactone. These mycobacteria—collectively known as mycolactone-producing mycobacteria (MPM)—have been given a variety of species names, including *Mycobacterium shinshuense*, *Mycobacterium pseudoshottsii*, *Mycobacterium marinum*, and *Mycobacterium* “liflandii”. Here we highlight the fact that all MPM share sufficient phenotypic and genotypic characteristics such that they should all be formally recognised as *M. ulcerans* and not separate species. Renaming all MPM as *M. ulcerans* is taxonomically correct and will resolve the confusion that is prevalent in the field and will assist political and financial advocacy for Buruli ulcer.

Defining a bacterial species has become an increasingly difficult task, particularly when bacteria exhibit different phenotypes but are genetically very closely related. Genomics has shown us very clearly that subtle genetic differences between bacteria can result in impressive phenotypic differences. It is not surprising that the expansion of bacterial genomics has led to a reassessment of the taxonomy of many bacterial species.

Such is the case with *M. ulcerans*, *M. marinum*, and other closely associated mycobacteria. *M. ulcerans* and *M. marinum* are genetically related species that cause quite different human skin diseases. *M. ulcerans* causes Buruli ulcer, a disease characterised by chronic and severe skin ulcers. The bacterium produces a lipid toxin called mycolactone, replicates slowly (doubling time >48 h) [1], and is apigmented. In contrast, *M. marinum* causes relatively minor granulomatous skin lesions, often referred to as “fish tank granulomas”, has a doubling time of 6–11 h, and produces bright yellow pigments when exposed to light. Despite their widely different phenotypes, genome comparisons have shown that these species share over 4,000 genes with 98.3% average DNA sequence identity [2]. However, there are also some important genetic differences between them. DNA–DNA hybridisation (DDH) analysis confirmed their status as distinct species, as inter-species relative hybridisation ratios (RBR) were less than 40% [3], [4]. The low RBR is explained by a number of features unique to *M. ulcerans*, such as the presence of a large virulence plasmid (pMUM) required for mycolactone production, and multiple copies of the insertion sequence element IS*2404* that itself accounts for 6% of the *M. ulcerans* genome [2], [5].

**Figure 1:**
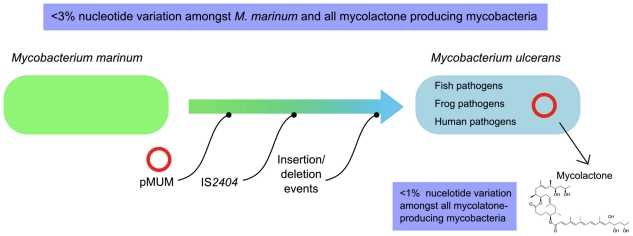
Overview of the evolution and principal species-defining features of *Mycobacterium ulcerans* as established by multi-locus sequence and genome deletion analysis.

Mycobacteria isolated recently from humans, fish, and frogs around the world (including Japan, the Mediterranean Sea, the Red Sea, Belgium, and the United States) have been variously called *M. shinshuense*, *M. marinum*, *M. pseudoshottsii*, or given unofficial names such as *M.* “liflandii” [6]–[10]. Subsequent studies have used the collective term MPM when describing *M. ulcerans* and these bacteria, as they all produce a form of mycolactone [5], . Phylogenetic studies of more than 50 *M. ulcerans*, other MPM, and *M. marinum* strains, based on multi-locus sequence analysis (MLSA) of chromosomal and pMUM sequences and studies of large DNA InDel polymorphisms, indicate that all MPM have likely evolved from a common *M. marinum* progenitor [5], [11], [12] and have then diverged again into two distinct lineages, with both lineages bearing strains that cause Buruli ulcer [5], [13] (Figure 1).

The new species assignations for MPM have not considered their genomic context and have been based on variable phenotypic characteristics (such as colony morphology and in vitro growth rates) and limited, monophyletic rRNA, *hsp65*, or *rpoB* analyses, which have shown these mycobacteria have a few unique nucleotide sequences when compared to a small number of allele sequences in GenBank. However, more complex and time-consuming DDH analyses, which, together with 16S rRNA sequencing, are the prescribed methods for defining a prokaryotic species [14], were not performed in these studies. In the only study to utilise DDH to investigate the relationship between recently described MPM and *M. marinum*, Yip et al. (2007) showed that MPM have an RBR of 88%–100% when compared to *M. ulcerans* strains from Africa and Australia and only 15%–60% RBR when compared with a genetically diverse range of nonmycolactone-producing *M. marinum* strains [5] (Table 1). Furthermore, the analysis of large sequence polymorphisms down to the exact nucleotide breakpoints also showed clear clustering of strains that have been assigned different species names, rendering these assignments inadequate [11].

**Table 1:** The Key Characteristics That Define *Mycobacterium ulcerans*.

Current Name	Source	RBR to *M. marinum* [5]	RBR^a^ to MU Agy99 [5]	High Copy IS*2404*	pMUM Plasmid	Mycolactone Produced (Type)	*M. ulcerans* (Yes/No)
*M. ulcerans* Agy99	Human clinical isolate, Ghana	52%	100%	+	+	+ A/B	Yes
*M. shinshuense* 753	Human clinical isolate, Japan	29%	94%	+	+	+ (A/B)	Yes
*M. pseudoshottsii* L15	Striped bass (*Morone saxatilis*), US	41%	98%	+	+	+ (F)	Yes
*M. marinum* CC240299	Koi (*Cyprinus carpio*), Israel	39%	100%	+	+	+ (F)	Yes
*M. marinum* DL240490	European sea bass (*Dicentrarchus labrax*), Red Sea	37%	91%	+	+	+ (F)	Yes
*M. marinum* DL045	European sea bass (*Dicentrarchus labrax*), Mediterranean Sea	32%	94%	+	+	+ (F)	Yes
*M*. “liflandii” 128FXT	African tropical clawed frog (*Xenopus tropicalis*), US	33%	100%	+	+	+ (E)	Yes
*M. marinum* M	Human clinical isolate, US	100%	52%	-	-	-	No

a RBR, relative binding ratio, derived from DNA–DNA hybridisation experiments.

A species is defined as “...a category that circumscribes a (preferably) genomically coherent group of individual isolates/strains sharing a high degree of similarity in (many) independent features, comparatively tested under highly standardized conditions” [15]. In practice, a prokaryotic species is considered to be a group of strains (including the type strain) that is characterised by a certain degree of phenotypic consistency, showing greater than 97% 16S rRNA gene-sequence identity and greater than 70% DDH [16]. If these criteria are applied to the MPM, all of which are “genomically coherent” as revealed by MLSA and InDel analysis, have >98% 16S rRNA identity to *M. ulcerans*, >70% DDH, possess pMUM plasmids, contain IS*2404*, and make mycolactone, they can clearly be considered as variants of the same species, namely *M. ulcerans*. It is on this solid genetic and phenotypic basis that we propose all MPM should be considered strains of *M. ulcerans*. Furthermore, we suggest that characteristics such as growth rate, colony morphology, pigment production, enzymatic activity, antibiotic susceptibility, and pathogenicity are useful traits for characterizing a particular mycobacterium, but are too sensitive for reliably defining a new taxon. Defining mycobacteria that satisfy our proposed diagnostic criteria as outlined in Table 1 as *M. ulcerans* will greatly simplify the nomenclature and alleviate confusion. It does not matter that under this revised naming scheme some strains of *M. ulcerans* will not be associated with human disease. Indeed, many MPM, such as *M. pseudoshottsii*, have only been associated with disease in animals other than humans; however, they still present the same consistent genetic signatures to assign them as strains of *M. ulcerans*. Furthermore, the extent of *M. ulcerans* recovered from humans to also cause disease in other animals, including koalas, possums, cats, and horses, is now being realised [17], [18]. These factors demonstrate how pathogenicity or host range of a bacterium is not a useful parameter for defining a species.

Reclassifying all MPM as *M. ulcerans* is more than an academic exercise. It will also highlight both the large geographic distribution and broad host range of this organism. Advocacy for a neglected tropical disease is not helped with confusion about the name of the causative organism. For example, renaming *M. shinshuense* to *M. ulcerans* would assist efforts to raise awareness about Buruli ulcer in Japan. Similarly, highlighting the fact that *M. ulcerans* is found around the world, including Europe and the US, can only help promote research in this field and encourage broader community interest in Buruli ulcer.
